# Association of the composition of the bone marrow tumor microenvironment in BCR::ABL1-negative myeloproliferative neoplasms with IFN-γ signaling and driver mutations

**DOI:** 10.1038/s41375-025-02706-3

**Published:** 2025-08-05

**Authors:** Marcus Bauer, Christoforos Vaxevanis, Nadja Jaekel, Hubert Hackl, Andreas Wilfer, Clara Zoellig, Monika Haemmerle, Carsten Müller-Tidow, Haifa Kathrin Al-Ali, Barbara Seliger, Claudia Wickenhauser

**Affiliations:** 1 Institute of Pathology, University Hospital Halle, Martin Luther University Halle-Wittenberg, Halle (Saale), Germany; 2 Medical Faculty, Martin-Luther-University Halle-Wittenberg, Halle (Saale), Germany; 3 Department of Hematology and Oncology, University Hospital Halle, Martin Luther University Halle-Wittenberg, Halle (Saale), Germany; 4 Institute of Bioinformatics, Biocenter, Medical University Innsbruck, Innsbruck, Austria; 5 Krukenberg Cancer Center Halle, University Hospital Halle, Martin Luther University Halle-Wittenberg, Halle (Salle), Germany; 6 Department of Hematology Oncology and Rheumatology, Heidelberg University Hospital, Heidelberg, Germany; 7 Fraunhofer Institute for Cell Therapy and Immunology, Leipzig, Germany; 8 Faculty of Health Sciences Brandenburg, Institute of Translational Immunology, Brandenburg Medical School “Theodor Fontane”, Brandenburg an der Havel, Germany; 9 Center of Translational Medicine, Brandenburg Medical School “Theodor Fontane”, Brandenburg an der Havel, Germany

**Keywords:** Immunosurveillance, Translational research

## Abstract

Constitutive JAK/STAT pathway activation is crucial in the pathogenesis of BCR::ABL1-negative myeloproliferative neoplasms (MPN), but has not yet been linked to interferon (IFN)-γ signaling and tumor microenvironment. Human *JAK2* V617F-mutated cell lines, 265 bone marrow biopsies (BMB) of two MPN cohorts, and 50 non-neoplastic BMB, revealed an intrinsic activation of IFN-γ signaling, which was confirmed by public RNA expression data. In vitro analysis of *JAK2*-mutated cell lines showed an activation of IFN-γ signaling pathway in the absence of IFN-γ in the cell supernatants. In addition, a heterogeneous, but increased expression of IFN-γ signaling components was found in BMB of *JAK2*-mutated samples with the highest expression in lymphocytes and monocytes, accompanied by increased tumor infiltrating lymphocytes (TIL). Unsupervised clustering identified a prognostic favorable cluster in both patient cohorts characterized by augmented IFN-γ signaling and TILs. This cluster was enriched with *JAK2*-mutated, JAK-inhibition naive MPN, mainly essential thrombocythemia and polycythemia vera with mild bone marrow fibrosis. Moreover, in silico data confirmed the link between *JAK2* mutations and increased IFN-γ signaling. Multivariate Cox regression revealed TILs to be the strongest prognostic marker. In conclusion, *JAK2*-mutated MPN exhibit an intrinsic activation of IFN-γ signaling associated with changes in the BM TME and patients’ outcome.

**Figure:**
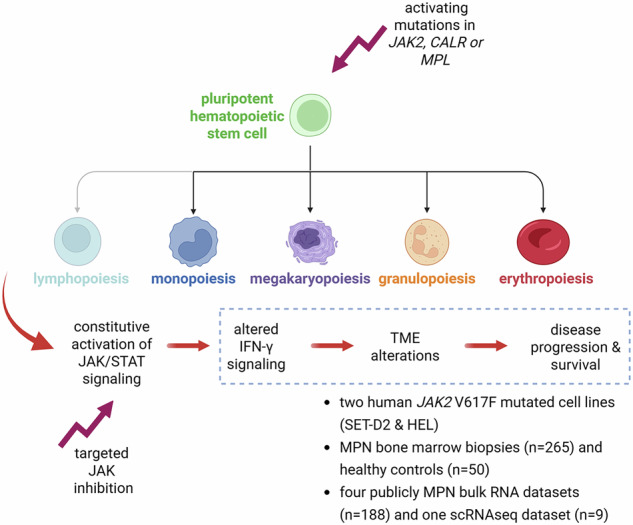
Constitutive activation of the Janus kinases and signal transducer and activator of transcription (JAK/STAT) signaling pathway mainly mediated by mutations in the *JAK2*, *CALR* and *MPL* genes in pluripotent hematopoietic stem cells (HSC) is crucial for the pathogenesis of BCR::ABL1-negative myeloproliferative neoplasms (MPN). Despite the activation of JAK/STAT signaling and its influence on the proliferation of malignant cells is well studied in patient samples and *JAK2-* and *CALR*-mutated cell systems, there exists limited information about the link between interferon (IFN)-γ signaling and bone marrow (BM) environment alterations. Therefore, two human *JAK2* V617F-mutated cell lines, 265 bone marrow biopsies (BMB) of MPN patients, separated in two independent cohorts, both with known clinical parameters, such as driver mutations, treatment and survival, 50 non-neoplastic BMB and five publicly available bulk and single cell RNA expression data sets of MPN samples with known *JAK2* or *CALR* mutation status were analyzed regarding (i) the role of IFN-γ signaling, (ii) its interrelation with the composition of the local BM tumor microenvironment (TME), (iii) the expression of immune response relevant molecules and (iv) their impact on patients’ survival. Created in BioRender. Bauer, M. (2025) https://BioRender.com/h133y7w.

Constitutive activation of the Janus kinases and signal transducer and activator of transcription (JAK/STAT) signaling pathway mainly mediated by mutations in the *JAK2*, *CALR* and *MPL* genes in pluripotent hematopoietic stem cells (HSC) is crucial for the pathogenesis of BCR::ABL1-negative myeloproliferative neoplasms (MPN). Despite the activation of JAK/STAT signaling and its influence on the proliferation of malignant cells is well studied in patient samples and *JAK2-* and *CALR*-mutated cell systems, there exists limited information about the link between interferon (IFN)-γ signaling and bone marrow (BM) environment alterations. Therefore, two human *JAK2* V617F-mutated cell lines, 265 bone marrow biopsies (BMB) of MPN patients, separated in two independent cohorts, both with known clinical parameters, such as driver mutations, treatment and survival, 50 non-neoplastic BMB and five publicly available bulk and single cell RNA expression data sets of MPN samples with known *JAK2* or *CALR* mutation status were analyzed regarding (i) the role of IFN-γ signaling, (ii) its interrelation with the composition of the local BM tumor microenvironment (TME), (iii) the expression of immune response relevant molecules and (iv) their impact on patients’ survival. Created in BioRender. Bauer, M. (2025) https://BioRender.com/h133y7w.

## Introduction

BCR::ABL1-negative myeloproliferative neoplasms (MPN) comprise a heterogeneous group of hematological neoplastic diseases that is characterized by cytosis of one or more cell lineages in the peripheral blood, associated with a significant risk of developing a secondary acute myeloid leukemia (sAML) [1]. Three subtypes, namely essential thrombocythemia (ET), polycythemia vera (PV), and primary myelofibrosis (PMF), can be distinguished based on molecular, histomorphological and clinical criteria of the World Health Organization [2]. They all share a constitutive activation of the Janus kinases and signal transducer and activator of transcription (JAK/STAT) signaling pathway that is crucial for the initiation and progression of MPN [3]. Therefore, targeted therapies with JAK inhibitors (JAKi) were developed over the last two decades. The JAK1/JAK2 inhibitor Ruxolitinib was approved in 2011 and the JAK2/FLT3 inhibitor Fedratinib in 2019 by US Food and Drug Administration (FDA) for treatment of PMF [4]. Lately, also Momelotinib was approved [5]. While the phosphorylation of STAT3 and downstream effectors are assumed to be most important in the pathogenesis of MPN [6], the JAK/STAT signaling pathway also alters the interferon (IFN)-γ signaling that is essential for an adequate host immunity [7]. Under physiological conditions, IFN-γ is secreted by activated T cells and NK cells [8] due to their stimulation by interleukins [9] or pathogens [10]. Recently, there exists increasing evidence for a dual function of IFN-γ in acute and chronic signaling. While IFN-γ exerts direct cytotoxic effects in acute inflammatory conditions, it also exhibits an immune suppressive activity in chronic inflammation accompanied by changes in the composition of the tumor microenvironment (TME) that were associated with cancer progression. Chronic IFN-γ signaling inhibits the maintenance, clonal diversity and activity of T cells, which leads to a decreased anti-tumor immunity [11]. Vice versa, the inhibition of the IFN-γ signaling resulted in phenotypically less exhausted tumor-infiltrating lymphocytes (TILs), but an impaired immune checkpoint (ICP) inhibition [12, 13]. Since IFN-γ has been demonstrated to regulate many survival and apoptotic pathways, but also the expression of different immune response relevant molecules, like classical human leukocyte antigen class I (HLA-I) and non-classical HLA-Ib antigens [14, 15], chemokines and ICP molecules, such as programmed cell death ligand 1 (PD-L1) [16] or cytotoxic T lymphocyte-associated protein 4 (CTLA4) [17], it exerts distinct biological consequences highlighting the pleiotropic activities of this cytokine. Thus, IFN-γ can generate anti-tumoral responses or enhance pro-tumoral signaling events dependent on the immune signature of tumors.

Since the functional consequences and clinical relevance of the IFN-γ signaling has not yet been analyzed in MPN, this study analyzed the local IFN-γ expression and IFN-γ signaling in cell supernatants and the BM TME in (i) two human *JAK2* V617F-mutated cell lines in vitro, (ii) in BMB of 265 MPN patients with known driver mutations and clinical parameters including patients’ outcome as well as 50 non-neoplastic BM (nnBM) cases, (iii) its correlation to the immune cell composition of the bone marrow (BM) TME and (iv) expression of immune response relevant factors, like ICP molecules and HLA-I. These data were associated to the mutational profile, therapy and patients’ outcome and further validated by analysis of five different publicly available data sets of MPN samples and healthy controls.

## Materials and methods

### Cell lines and cell culture

The cell line SET-2 was purchased from the German Collection of Microorganisms and Cell Cultures GmbH (Hannover, Germany), while the HEL cell line was kindly provided by the research laboratory of the Department of Pediatrics, University Hospital Halle, Germany. Both cell lines were cultured in RPMI-1640 supplemented with 10% fetal calf serum (FCS) and 1% antibiotics.

### Cytokine secretion assay

The cytokine concentrations in cell supernatants were determined by the LEGENDplex™ Essential Immune Response Panel (Biolegend, California, USA) using a BD Fortessa flow cytometer according to manufacturer’s instructions.

### Western blot analysis

For the quantification of proteins, total protein was extracted from cell pellets and quantified using the Pierce™ BCA Protein Assay Kit (Thermo Fisher, Waltham, MA, USA). Ten to 25 μg total protein/lane was subjected to Western blot analysis as recently described [18] using specific antibodies (Abs) listed in Supplementary Table S1.

### RNA isolation, RNA sequencing and data analysis

Total cellular RNA was isolated with NucleoSpin RNA Plus, Mini kit for RNA purification with DNA removal column (Macherey-Nagel, Düren, Germany) according to the manufacturer’s instructions. For RNA sequencing (RNAseq), 2 μg of total RNA/sample was employed and strand specific 150 base paired-end RNAseq was performed using the Illumina NovaSeq platform by Genewiz (Leipzig, Germany). Approximately 20 million reads per sample were obtained. Reads were trimmed using Trimmomatic, quality checked with fastqc 0.11.9 and mapped to the human reference genome (hg38) employing the splice aware aligner STAR 2.7.9a. Quantifications on NCBI gene models (hg38refGene) were performed using featureCounts v2.0.0.

### Ethics approval and consent to participate

The scientific use of the FFPE BMB was approved by the Ethical Committee of the Medical Faculty, Martin Luther University Halle-Wittenberg, Germany (2017-81 and 2023-196). Informed consent was obtained from all participants. All methods were performed in accordance with the relevant guidelines and regulations.

### MPN and HC patients’ samples

Formalin fixed and paraffin embedded (FFPE) BMB were collected between 2014 and 2023 and archived at the Institute of Pathology of the University Hospital Halle, Martin Luther University Halle-Wittenberg, Germany. The collective encompasses 50 non-neoplastic BM (nnBM) serving as age-matched HCs and 265 MPN samples. The MPN samples were separated in a test (*n* = 152) and a validation cohort (*n* = 113) summarized in Table 1. Clinical data from these patients were available, such as age, sex, disease status, therapy and survival time (Table 1). Progression-free survival (PFS) data of MPN patients were obtained with eight years follow up and referred to progression to sAML, myeloid sarcoma manifestation or disease related death. Clinical risk was assessed using the DIPPS score for PMF, European LeukemiaNet stratification for ET, and the IPSS for PV [19–21].

**Table 1:** Clinical and pathological characteristics of the test and validation MPN cohort.

Cohort			Controls	Test cohort			Validation cohort		
Category			HC (nnBM)	ET & post-ET-MF	PV & post-PV-MF	PMF	ET & post-ET-MF	PV &. post-PV-MF	PMF
Number of samples			50	43	39	70	23	24	66
Sex	male	*n* (%)	29 (58.0)	22 (53.7)	18 (43.9)	40 (57.1)	9 (39.1)	14 (58.3)	41 (62.1)
	female	*n* (%)	21 (42.0)	19 (46.3)	23 (56.1)	30 (42.9)	14 (60.9)	10 (41.7)	25 (37.9)
Age (years)		range (mean)	20-87 (62.1)	35-92 (67.9)	35-87 (65.7)	32-84 (61.6)	44-82 (63.8)	48-72 (61.5)	33-85 (60.6)
MF	0	*n* (%)	50 (100)	20 (48.8)	19 (46.3)	4 (5.7)	7 (30.4)	4 (16.6)	5 (7.6)
	1	*n* (%)	0	12 (29.3)	8 (19.5)	13 (18.6)	4 (17.4)	9 (37.5)	8 (12.1)
	2	*n* (%)	0	6 (14.6)	8 (19.5)	25 (35.7)	5 (21.8)	8 (33.3)	29 (43.9)
	3	*n* (%)	0	3 (7.3)	6 (14.6)	28 (40)	7 (30.4)	3 (12.6)	24 (36.4)
JAKi	No	*n* (%)	-	38 (92.7)	35 (85.4)	56 (80)	11 (47.8)	17 (70.8)	34 (51.5)
	Yes	*n* (%)	-	3 (7.3)	6 (14.6)	14 (20)	12 (52.2)	7 (29.2)	32 (48.5)
Mutation	*JAK2*	*n* (%)	-	28 (65.1)	39 (100.0)	42 (60.0)	11 (47.8)	24 (100)	37 (56.1)
	*CALR*	*n* (%)	-	7 (16.3)	0 (0.0)	18 (25.7)	12 (52.2)	0 (0.0)	16 (24.2)
	*MPL*	*n* (%)	-	0 (0.0)	0 (0.0)	3 (4.3)	0 (0.0)	0 (0.0)	9 (13.6)
	TN	*n* (%)	-	8 (18.6)	0 (0.0)	7 (10.0)	0 (0.0)	0 (0.0)	4 (6,1)
Follow-up	no event	*n* (%)	-	17 (41.5)	15 (36.6)	44 (62.9)	20(86.9)	16 (66.7)	47 (71.2)
	event	*n* (%)	-	7 (17.1)	9 (22)	20 (28.6)	3 (13.1)	8 (33.3)	19 (38.8)
	unknown	*n* (%)	-	17 (41.5)	17 (41.5)	6 (8.6)	0 (0.0)	0 (0.0)	0 (0.0)

*ET* essential thrombocythemia, *HC* healthy control, *MF* myelofibrosis, *nnBM* non-neoplastic bone marrow, *PMF* primary myelofibrosis, *PV* polycythemia vera, *TN* triple negative.

### Standard morphological evaluation of the bone marrow and conventional immunohistochemistry

Histopathological diagnosis was performed according to the diagnostic criteria of the World Health Organization (WHO) classification of Tumors of Hematopoietic and Lymphoid tissues, fourth edition 2017 and 2022 [2]. The degree of myelofibrosis was analyzed using Gomoris’ silver staining, and a four-fold scoring system was employed (MF-0 to MF-3) [2, 3].

For conventional immunohistochemistry (IHC), all BMB were stained with Abs directed against CD34, CD71, CD117, HLA-I HC, HLA-E, HLA-F, HLA-G, IRF1, lysozyme, MPO, NF-kB, OAS1, pJAK2, pSTAT1 and pSTAT3 according to the supplier’s instructions, which are summarized in Supplementary Table S1. Further information of MPN diagnosis was taken from the Medical Records including cytology, mutational status and peripheral blood parameters.

### Multispectral imaging

To analyze the spatial immune cell distribution of different immune cell subpopulations and the expression of ICP molecules, multispectral imaging (MSI) was performed as recently described employing six different multiplex Ab panels as listed in Supplementary Table S2 [22]. Briefly, after antigen retrieval, the tissues were incubated for 30 min with the primary Ab followed by the secondary Ab (Akoya Biosciences, Marlborough, MA, USA, Opal Polymer HRP Ms + Rb) for 10 min. Tyramide signal amplification (TSA) visualization was performed using the Opal seven-color IHC kit (Opal 520, Opal 540, Opal 570, Opal 620, Opal 650, Opal 690, Akoya Biosciences) and DAPI. Stained slides were imaged employing the PhenoImager HT platform (Akoya Biosciences). Cell segmentation and phenotyping were performed using the inForm software (Akoya Biosciences). The frequency of immune cell populations and their cartographic coordinates were evaluated using the R packages phenoptr and phenoptrReports packages (https://github.com/akoyabio/phenoptrReports).

#### In-situ hybridization

The IFN-γ expression of MPN and nnBM samples was determined by in-situ hybridization (ISH) employing a probe directed against human *IFNG* mRNA and combined with an IHC for CD3 (Supplementary Table S1) using the RNAscope Multiplex Fluorescent V2 Assay (Bio-Techne, Minneapolis, MI; USA) according to manufacturer’s instructions. Briefly, after antigen retrieval with pH6, the tissues were incubated for 30 min with a primary anti-CD3 Ab followed by a washing step and incubation with an *IFNG* C1 probe (Bio-Techne) for 2 h. RNAscope Multiplex FL v2 AMP1-3 and HRP-C1 were applied according to manufacturer’s instructions. The *IFNG* signal was visualized with Opal 540 (Akoya Biosciences), while HRP was blocked with the RNA scope Multiplex FL v2 HRP blocker. To visualize the CD3 staining, a HRP-linked secondary Ab (Akoya Biosciences, Opal Polymer HRP Ms + Rb) was added for 10 min. Tyramide signal amplification (TSA) visualization was performed with Opal 690 (Akoya Biosciences) followed by counterstaining with DAPI (Akoya Biosciences). The expression levels of *IFNG* were analyzed by employing the inform software (Akoya Biosciences). The mean fluorescence intensity (MFI) and the percentage of positive cells were calculated.

### IFN-γ signaling pathway analysis of publicly available data sets

Components of the IFN-γ signaling pathway were identified by employing the open-source, peer-reviewed pathway database reactome (https://reactome.org, ID: R-HSA-877300.6) [23]. After downloading of the normalized gene expression data from Gene Expression Omnibus (https://www.ncbi.nlm.nih.gov/geo/) IFN-γ signaling pathway component expression was analyzed in the four publicly available data sets GSE53482, GSE103176, GSE174060 and GSE206768 comprising healthy controls (HC) and MPN samples, as well as *CALR*-mutated MARIMO cells (PRJNA804684) summarized in Table 2.

**Table 2:** Features of the publicly available RNA expression data sets analyzed.

Gene set	Number of samples	Cell/tissue type	RNA expression data type	Reference
GSE53482	42 PMF 31 HC	Peripheral blood CD34^+^ cells	Affymetrix HG-U219	[24]
GSE103176	24 ET, 26 PV 15 HC	Peripheral blood CD34^+^ cells	Affymetrix HG-U219	[25]
GSE174060	6 ET 11 PV 9 PMF 4 secondary myelofibrosis 6 HC	Peripheral blood CD34^+^ cells	Affymetrix Human Transcriptome Array 2.0	[26]
GSE206768	29 PMF	Bone marrow (not sorted for CD34^+^ cells)	249 immune genes Nanostring nCounter GX Human Inflammation panel	[27]
PRJNA804684	3 biological replicates	MARIMO cells	RNAseq data	[28]
ZENODO 10.5281/zenodo.12708342	6 MPN (untreated) 3 HC	Bone marrow (not sorted for CD34^+^ cells)	scRNAseq data	[29, 30]

Differential gene expression of different groups (MPN versus HC or *JAK2*-mutated versus no *JAK2* mutation) was performed using PyDESeq2 package in Python [24].

Gene set enrichment analysis (GSEA) was performed using prerank function of GSEApy [25] and the MSigDB_Hallmark_2020 gene set (https://www.gsea-msigdb.org/gsea/msigdb/collections.jsp) [26, 27]. Differentially expressed genes were not filtered for statistical significance before ranking. The normalized enrichment score (NES) and the false discovery rate (FDR) were calculated for all gene sets. In addition, the RNA data set GSE206768 containing 29 BM PMF samples with known degree of myelofibrosis (MF-0 versus MF-3) available RNA expression data of 249 immune genes (Nanostring nCounter GX Human Inflammation panel) was employed [28]. Cell line derived gene expression data from MONO-MAC-6, NB4, HL-60, K052, NOMO-1, SKM-1, TF-1, TUR, U-937, KG-1, MOLM-13, MV-4-22 and SIG-M5 were achieved from Cell Model Passports (https://cellmodelpassports.sanger.ac.uk/) and the Expression Atlas (https://www.ebi.ac.uk/gxa/experiments/E-MTAB-2770/Results) [29]. RNA expression data were used for single sample GSEA (ssGSEA) employing the ssGSEA extension of GSEApy [30]. In addition, RNA expression data of MARIMO cells [31] were compared to that obtained from *JAK2*-mutated HEL and SET-2 cells (three biological replicates each) using PyDESeq2 package in Python. The single cell RNA-seq (scRNAseq) data [32, 33] encompassing three HC and six untreated MPN was analyzed using R (version 4.5.0). The Seurat object containing harmonized data was downloaded from 10.5281/zenodo.12708342 and analyzed to explore gene expression. Dimensionality reduction results were visualized using UMAP, the cells were grouped by Seurat clusters or annotated cell types. Expression of genes was visualized across the UMAP embedding using Seurat’s FeaturePlot function. Differential gene expression between MPN and HC was conducted using Seurat’s FindMarkers function. GSEA was then performed for each cell type using the fgsea package and Hallmark gene sets from MSigDB, based on ranked log2 fold-change values. The differentially expressed pathways were visualized using ggplot2, and significant results (adjusted *p*  <  0.05) were shown across different cell types to generate a summary dot plot.

### Statistics

The Mann-Whitney U test was employed to compare two variables. Multiple variables were analyzed by using a one-way ANOVA. Linear correlations were estimated using Pearson’s correlation. The heatmaps were created using Pandas package (https://github.com/pandas-dev/pandas) in Python [34], data normalization was done using StandardScaler. Survival analyses were performed on 112 patients of the test cohort and 113 patients of the validation cohort (follow-up time up to 96 months) using Kaplan-Meier estimators, log-rank tests and Cox regression models. *P* values < 0.05 were considered statistically significant. Figures were prepared using the GraphPad Prism 7.0 software, IBM SPSS Statistics 28.0, and Python.

## Results

### Clinical presentation of MPN patients

In total, 265 BMB from MPN patients separated in a test (*n* = 152) and a validation cohort (*n* = 113), as well as 50 nnBM serving as HCs were analyzed. Clinical parameters and the mutational profile are summarized in Table 1. The mean age of the MPN cohort was 64 years, and 44.6% of these patients were female, while the mean age of the HC cohort was 62 with 42.0% female donors. MPN BMB showed no or only mild myelofibrosis (MF 0-1) in 42.6%. In MPN BMB, mutations in the *JAK2* gene were found in 68.3%, mutations in *CALR* and *MPL* genes in 20.0% and 4.5% of cases, respectively. No mutations in the *JAK2*, *CALR* or *MPL* genes were identified in the BMB of 19 patients, which were referred to triple negative (TN) cases. From 265 patients, 74 (27.9%) were previously treated with JAKi. In an 8-year follow-up period, 71/221 patients (32.1%) showed an event (progression to sAML, manifestation of myeloid sarcoma or disease-related death).

### Altered IFN-y signaling in the human JAK2 V617F-mutated cell lines and BMB of MPN compared to nnBM

**Fig. 1:**
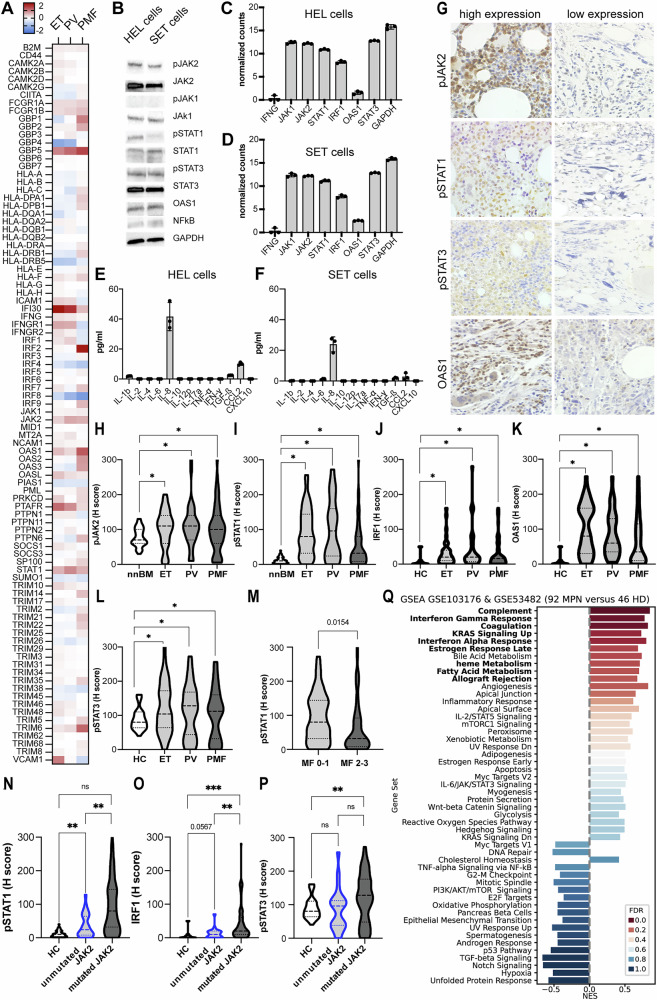
Increased IFN-γ signaling in BMB of MPN compared to healthy controls. **A** Expression analysis of 91 IFN-γ-regulated genes in publicly available RNA expression data sets (GSE53482 and GSE103176) of ET, PV and PMF compared to HC is shown in a heatmap. The 91 genes were selected based on the open-source, peer-reviewed pathway database reactome (https://reactome.org, ID: R-HSA-877300.6). Upregulated genes are highlighted in red, and downregulated genes are marked in blue. The color scheme is given on the right side of the graph. **B** Constitutive expression of pJAK2, JAK2, pJAK1, JAK1, pSTAT1, STAT1, pSTAT3, STAT3, OAS1 is shown in untreated HEL and SET-2 cells. **C**, **D** RNA expression of HEL and SET-2 cells (three biological replicates) presented as counts of the housekeeping gene *GAPDH* and *IFNG*, *JAK1*, *JAK2*, *STAT1*, *STAT3* as a bar plot. **E**, **F** Cytokine expression assay of *JAK2* V617F-mutated HEL and SET-2 cell supernatants and data are presented as pg/ml. **G** A representative IHC staining for selected IFN-γ pathway components. Immunohistochemical analyses of consecutive tissue slides were performed to determine the expression of IFN-γ and JAK/STAT signaling pathway (pJAK2, pSTAT1, pSTAT3, and OAS1) components. A positive staining was found in various cell subsets including hematopoietic cells, e.g., megakaryocytes and myelopoietic precursors. **H**–**L** Expression of IFN-γ pathway components was analyzed by IHC, and data are presented as H scores. Results in the different MPN subtypes and in nnBM are depicted with violin plots. Significant differences are marked with asterisks (*p* < 0.05). **M** pSTAT1 expression in MPN BM with no or only low BM fibrosis (MF 0-1) to that with advanced BM fibrosis (MF 2-3). **N**–**P** The expression of selected IFN-γ pathway components in MPN patients with *JAK2* versus *CALR* mutations. **Q** Gene set enrichment analysis (GSEA) was performed and demonstrating that the second most upregulated gene set in MPN samples compared to HC was genes associated to IFN-γ response (MSigDB_Hallmark_2020). The NES is given as the value of the bars, and the FDR is depicted in different colors. Gene sets with a significant up-/down-regulation are highlighted with bold letters.

Bioinformatics analyses using open-source database reactome [23] identified 91 genes involved in IFN-γ signaling. 18/91 genes exhibited a high expression in healthy BM and 24/91 genes in lymphoid tissues (Supplementary Figure S1A) including HLA-I antigens (*B2M*, *HLA-A*, *-B*, *-C*), HLA class II (HLA-II) antigens (*HLA-DP*, *-DQ and –DR)*, non-classical HLA-Ib antigens (*HLA-E*, *-F and -G*) and components of the IFN-γ signaling pathway (*JAK1*, *STAT1* and *IFNGR*) (Fig. 1A). Since *JAK2* mutations in MPN are accompanied by an activated JAK/STAT signaling [3], two human MPN cell lines with known *JAK2* V617F mutation were analyzed for the expression and activity of IFN-γ signaling components. As expected, both cell lines expressed high levels of pJAK2, pSTAT3, pSTAT1 and OAS1 (Fig. 1B). RNAseq analyses showed no *IFNG* mRNA expression, but high expression levels of the IFN-γ pathway components *JAK1*, *JAK2* and *STAT3* and lower expression levels of *STAT1* and *IRF1* (Fig. 1C, D). Analysis of the supernatants of HEL and SET-2 using a 13-plex cytokine array confirmed RNAseq data and demonstrated a lack of IFN-γ secretion, but high concentrations of interleukin-8 (IL-8) and low amounts of TGF-β and CCL2 (Fig. 1E-F). Furthermore, analyses of the IFN-γ signaling gene set expression in HEL and SET-2 cells as well as in 13 other hematological cell lines lacking *JAK2* V617F mutations identified the highest NES score in the SET-2 cell line (Supplementary Figure S1B). Moreover, GSEA comparing *JAK2*-mutated HEL and SET-2 cells with *CALR*-mutated MARIMO cells revealed an upregulation of various immunological pathways, including the IFN-γ signaling in the *JAK2*-mutated cell systems (Supplementary Fig. S1C). Using conventional IHC, pJAK1, pJAK2, pSTAT1, pSTAT3 and OAS1 protein expression was determined in 152 MPN samples (test cohort) and compared to that of 50 nnBM as representatively shown in Fig. 1G. We found a positive staining of these markers in different cell subsets, including hematopoietic cells, e.g., megakaryocytes and myelopoietic precursors. BMB of all MPN subtypes expressed significant higher levels of pJAK2, pSTAT1, IRF1 and OAS1 than the nnBM specimen (Fig. 1H–L). In addition, the expression of pSTAT1 and OAS1, but not of IRF1 was dependent on the MF grade (Fig. 1M, Supplementary Fig. S1D–G). However, it must be taken into account that the histologic assessment of BM samples with overt fibrosis and decreased levels of pSTAT1 and OAS1 showed a leakage of hematopoietic cells. Furthermore, the expression levels of these markers in the BM of *JAK2*- and *CALR*-mutated samples were investigated. Despite comparable allele frequencies of mutated *JAK2* and *CALR* genes, significantly higher pSTAT1, IRF1, and OAS1 expression was found in samples with JAK2 mutations. (Figure N-P, Supplementary Figure S1H-J).

Next, the link between the expression levels of the analyzed IFN-γ-regulated genes was validated in public available RNAseq data sets comparing CD34^+^ cells from MPN patients and HC. In total, 9981 significantly up- and 7208 downregulated genes were found in MPN compared to HC with a significant upregulation of the IFN-γ response genes compared to HC (Fig. 1Q, Supplementary Fig. S1K). Analysis of the public data set GSE206768 containing PMF samples with known MF grade revealed a significant upregulation of 42 genes (*p* < 0.05) including e.g., IL-8 and TGF-ß in patients with MF-3 versus MF-0 (Supplementary Fig. S2A) and genes related to IFN-γ response in PMF samples with high myelofibrosis (MF-3) compared to low myelofibrosis (MF-0) (Supplementary Fig. S2B, C). This upregulation of IFN- γ response-related molecules was not associated with increased IFNG mRNA expression in MF-3 samples, but showed increased expression of CD4 mRNA (Supplementary Fig. S2D, E). No correlation of *IFNG* mRNA expression and *STAT1*, *STAT3* or *CD4* mRNA was found in the GSE206768 data set (Supplementary Fig. S2F–H). Moreover, a significantly increased expression of the IFN-γ response gene set was detected in MPN patients with *JAK2* mutations compared to MPN patients with *CALR* mutations in the gene set GSE174060, while in gene set GSE103176 only a trend of higher IFN-γ was found (Supplementary Fig. S3A–H).

### Immune cell infiltration and local IFN-γ expression in MPN subtypes compared to nnBM

**Fig. 2:**
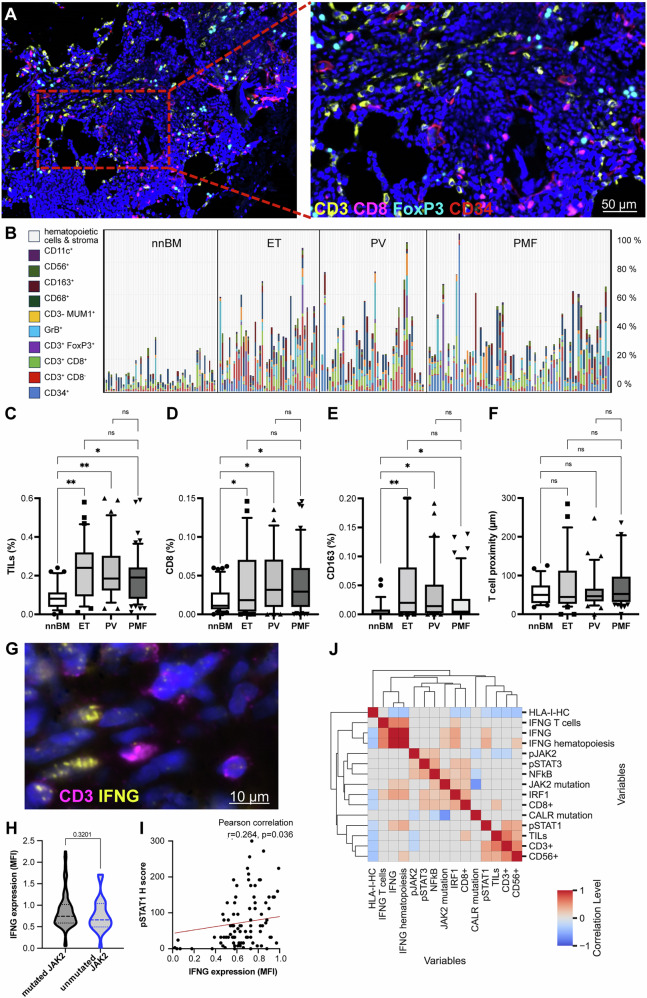
Immune cell composition in MPN BM. **A** Representative multiplex IHC of a BM sample from a patient with PMF with low BM fibrosis (MF-1). The amount and the composition of tissue infiltrating lymphocytes (TILs) was analyzed by MSI with a six-color Ab panel directed against CD34 (red), CD3 (yellow), CD8 (magenta), FOXP3 (turquoise), MUM1p (not shown), GrB (not shown) and counterstained with DAPI (blue). Consecutive slides were stained with Abs directed against PD-L1, PD-L2, gal-9, TIM-3, CTLA4, CD80, CD28, LAG3, CD56, CD68, CD163, and CD11c. Differences in the frequencies of immune cell subpopulations in 50 nnBM and 152 MPN samples (ET *n* = 43, PV *n* = 39, PMF *n* = 70) are depicted as a bar plot (**B**) and as box plots separately for (**C**) TILs (%), (**D**) CD8^+^ T cells (%), (**E**) CD163^+^ macrophages (%), and (**F**) the spatial proximity of CD3^+^CD8^-^ and CD3^+^CD8^+^ T cells (µm). Significant differences are marked with asterisks (* *p* < 0.05; ** *p* < 0.001). **G** Combined ISH with a probe directed against IFNG mRNA (yellow) and an IHC for CD3 (magenta). *IFNG* mRNA can be found in both CD3^+^ and CD3^-^ cells. **H** Violin plot showing the IFNG mRNA expression in MPN BM depending on the underlying driver mutation. **I** Scatter plot depiction of the association of IFNG mRNA expression and pSTAT1 expression in the BM. **J** Cluster correlation map of the IFNG expression with JAK/STAT signaling pathway components and the immune cell composition in the BM. Pearson correlation coefficients are displayed by different colors defined in the scale bar, and only statistically significant correlations (*p* < 0.05) are depicted.

Since the IFN-γ signaling might affect the composition of immune cells in the TME and vice versa, both the frequencies and the spatial distribution of CD3^+^CD8^-^ T cells, CD3^+^CD8^+^ T cells, CD3^+^FOXP3^+^ regulatory T cells (Tregs), CD3^-^MUM1^+^ B cells/plasma cells, CD3^+^ GrB^+^ T cells, CD3^-^GrB^+^ cells, CD11c^+^ dendritic cells (DCs), CD68^+^CD163^-^ M1 macrophages, CD68^+^CD163^+^ M2 macrophages and CD3^-^CD56^+^ (including CD56^+^CD16^+^) NK cells were determined in 202 BMB (test cohort and nnBM) using MSI (Fig. 2A). The mean TIL frequency was significantly increased in all MPN subtypes compared to nnBM. This applies in particular to higher numbers of CD8^+^ T cells, Tregs and CD163^+^ macrophages (Fig. 2B–E and Table 3). The spatial distribution of T cells revealed a comparable mean T cell proximity between CD3^+^CD8^+^ and CD3^+^CD8^-^ T cell subsets in nnBM and respective MPN subtypes (Fig. 2F). Analysis of the local *IFNG* mRNA expression in BMB along with CD3 as T cell marker using ISH (Fig. 2G) identified no significant difference of the *IFNG* mRNA expression in *JAK2-* versus *CALR-mutated* cases (Fig. 2H). However, Pearson correlation revealed a positive correlation with pSTAT1 and IRF1 expression as well as the presence of mutations in the *JAK2* gene (Fig. 2I, J). Furthermore, the presence of *JAK2* gene mutations was accompanied by higher IRF1 and NF-kB expression, while MPN patients with *CALR* gene mutations showed lower NF-kB and pJAK2 expression (Fig. 2J and Supplementary Fig. S4).

**Table 3:** Frequencies of different immune cell subsets in nnBM and different MPN entities.

Variable		HC (nnBM)				ET				PV				PMF				$x$^2^ test
	Value	Min		Max	Mean	Min		Max	Mean	Min		Max	Mean	Min		Max	Mean	p
TIIC	%	0	-	65	13.79	3.00	-	67.00	24.97	1.00	-	60.00	21.55	1.00	-	81.00	19.09	0.413
T cell	%	0	-	41.9	6.491	0.72	-	35.15	11.52	0.31	-	36.07	9.41	0.95	-	30.11	8.41	0.424
CD8	%	0	-	39.7	3.532	0.00	-	23.34	4.50	0.00	-	24.18	4.58	0.00	-	17.17	4.28	0.467
Treg	%	0	-	12	0.722	0.00	-	97.66	3.35	0.00	-	38.67	1.62	0.00	-	47.44	1.84	0.562
GrB	%	0	-	38.8	4.672	0.00	-	64.98	6.10	0.00	-	22.49	4.50	0.00	-	33.60	4.40	0.473
MUM1	%	0	-	13.1	1.154	0.00	-	9.67	1.74	0.00	-	37.85	2.14	0.00	-	73.54	2.27	0.470
CD11c	%	0	-	6	0.209	0.00	-	6.05	1.28	0.00	-	1.86	0.49	0.00	-	4.00	0.55	0.424
CD163	%	0	-	46.4	1.981	0.00	-	28.80	5.35	0.00	-	41.37	4.89	0.00	-	35.40	2.91	0.366
CD68	%	0	-	90.2	7.356	0.20	-	36.32	6.70	0.11	-	35.52	7.62	0.00	-	48.19	7.29	0.418
T cell distance	µm	15.83	-	588	78.89	0.00	-	357	80.09	0.00	-	248	62.54	0.00	-	357	83.05	0.455

*ET* essential thrombocythemia, *HC* healthy control, *nnBM* non-neoplastic bone marrow, *PMF* primary myelofibrosis, *PV* polycythemia vera, *TIIC* tumor infiltrating immune cells (T and B cell subsets).

### Cell type specific IFN-γ signaling in MPN

**Fig. 3:**
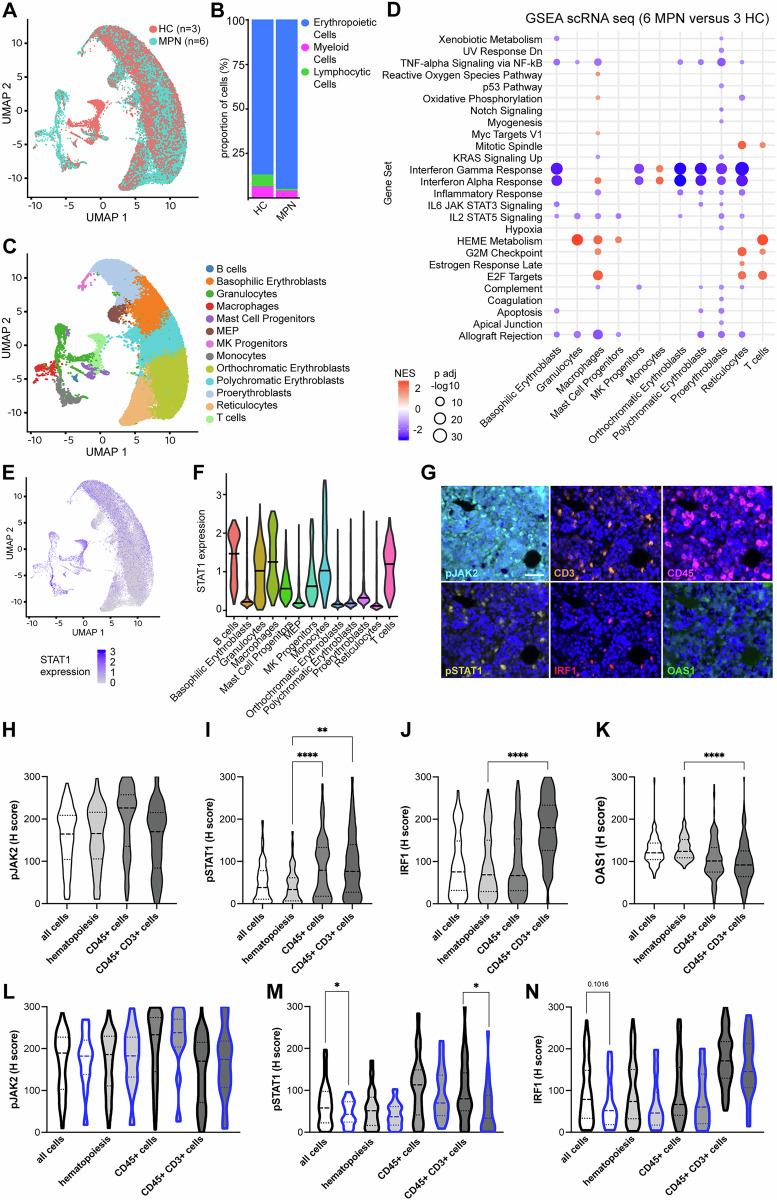
Cell type specific expression of IFN-γ signaling components in MPN. **A** Cells of scRNAseq data sets of HC and MPN depicted as UMAP. **B** Bar plot representing the distribution of annotated broad cell types in HC and MPN. **C** Annotated cell types in the scRNAseq data sets of HC and MPN depicted as UMAP. **D** GSEA in specific cell types of MPN samples compared to HC. Only significantly differentially expressed gene sets are depicted, and the normalized enrichment score (NES) and the *p*-value are given in the legend. **E** STAT1 expression in the data set shown as a UMAP and as violin plots **F**. **G** Representative multiplex IHC of a BM sample from a patient with PMF showing the expression of pJAK2 (turquoise), CD3 (orange), CD45 (magenta), pSTAT1 (yellow), IRF1 (red) and OAS1 (green). **H**–**K** The expression of selected IFN-γ pathway components in MPN patients in all cells, hematopoietic cells, CD45^+^ cells and CD45^+^CD3^+^ cells. Significant differences are marked with an asterisk (**p < 0.001; ***p < 0.0001). **L**–**N** The expression of pJAK2, pSTAT1 and IRF1 in all cells, hematopoietic cells, CD45^+^ cells and CD45^+^CD3^+^ cells depending on the driver mutation (black border indicate a mutation in *JAK2* and blue borders indicate mutations in *CALR*, *MPL* or triple negative cases).

To get insights in the cell type specific expression of IFN-γ signaling components, publicly available scRNAseq data were analyzed (10.5281/zenodo.12708342). Cells were grouped by conditions HC and MPN and showed in both groups high frequencies of erythropoietic cells (Fig. 3A, B). Next, annotated cell types shown in Fig. 3C were used for cell type specific GSEA of MPN versus HC (Fig. 3D) and revealed a downregulation of IFN-γ signaling in erythropoietic cells, but an upregulation of this pathway in monocytes (Fig. 3E, F). High expression levels of STAT1 were found in B and T cells, as well as myeloid cells, like granulocytes, macrophages and monocytes. Next, we analyzed the cell type specific expression of STAT1, IRF1 in association with the cell type specific IFNG expression and found an increased IFN-γ signaling response to be independent from local IFNG expression in MPN (Supplementary Fig. S5). These data were confirmed in our BM samples by MSI as representatively shown in Fig. 3G with the highest expression of pSTAT1 and IRF1 in CD45^+^ cells, including CD45^+^CD3^+^ T cells (Fig. 3H–K). Comparison of wildtype and JAK2-mutated MPN samples revealed a higher expression of pSTAT1 and IRF1 in JAK2-mutated cases with the highest levels in CD45^+^CD3^+^ T cells (Fig. 3L–N).

### Correlation of different immune clusters with the survival of MPN patients

**Fig. 4:**
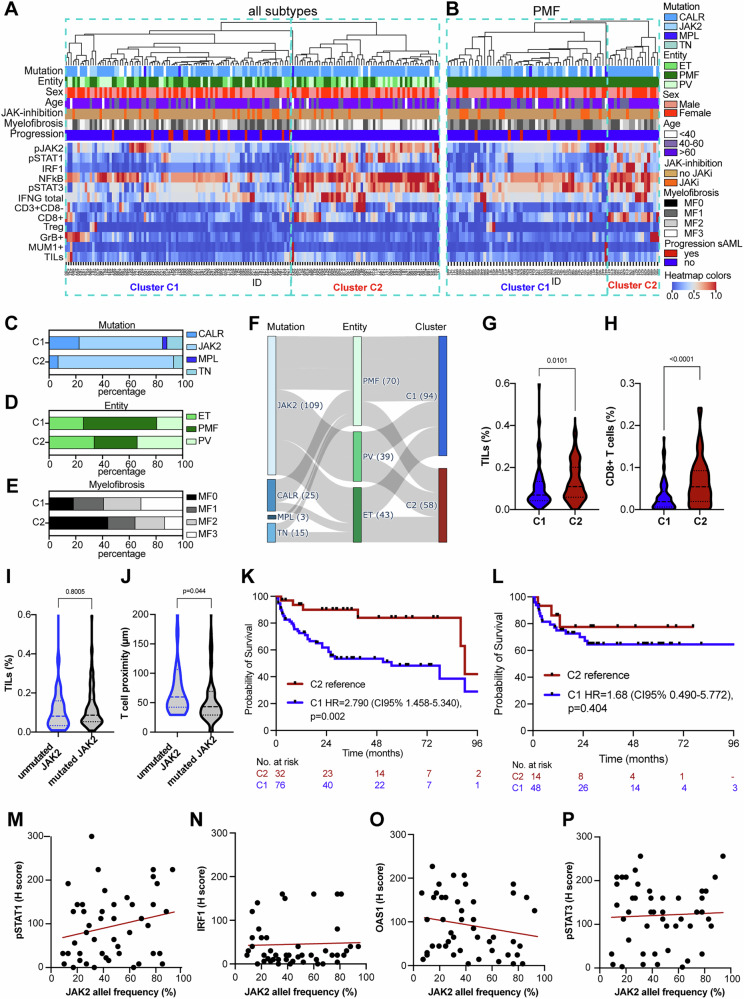
Interrelationship of the BM TME, MPN subtypes and survival. Unsupervised clustering based on the expression of components of JAK/STAT signaling, IFN-γ signaling, NF-kB and immune cell frequency is presented as a heatmap in all MPN subtypes (**A**) and PMF samples (**B**). Red tiles denote high expression, while blue tiles correspond to low expression (see color scheme heatmap at the right). The seven horizontal bars above the heatmap indicate the classification of samples according to driver mutation, MPN entity, sex, age, previous JAK-inhibition prior to BMB, myelofibrosis, and the progression to sAML in the follow up. The legend of the color labels is displayed on the right side of the heatmap. The clustering revealed two main clusters (C1-C2) based on the expression of the different factors. **C**–**E** Bar plots show the distribution of MPN entity, driver mutations and degree of myelofibrosis. **F** Sankey plot shows the interrelationship of driver mutations, MPN entity, and immune clusters. **G**, **H** Boxplots show the abundancy of TILs and CD8 + T cells in immune clusters C1 and C2. The *p*-value are given as numbers. **I**, **J** Boxplots show the abundancy of TILs and CD8 + T cells in JAK2-mutated versus non-JAK2-mutated MPN. The *p*-value are given as numbers. **K** Kaplan-Meier estimators illustrate the prognostic significance of TME-based clusters of MPN patients (all subtypes) and in PMF patients (**L**). **M**–**P** Scatter plots show the association of the allelic frequency of the mutated JAK2 gene and pJAK2, pSTAT1, AOS1 and pSTAT3 expression. The *p*-values are given as numbers.

Due to the prognostic relevance of several immune response relevant factors, an unsupervised clustering was used to identify whether an interrelationship between the expression of IFN-γ pathway components and the immune cell infiltration exists in all MPN subtypes or only in PMF, respectively (Fig. 4A, B). Based on the hierarchy visualized by the dendrogram, two major immune clusters (C1 and C2) were identified. The cluster C1 was characterized by lower expression of pSTAT1, pSTAT3, IRF1 and NF-kB compared to cluster C2. While the clusters showed no significant differences regarding age and sex of the patients, *JAK2*-mutated MPN were more present in cluster C2, which was associated with mainly JAKi-naive cases and an increased prevalence of PV and ET as well as lower BM fibrosis (Fig. 4C–F). Moreover, we found in average a higher infiltration by both TILs as well as CD8^+^ T cells in cluster C2 independent of the underlying driver mutation (Fig. 4G–J). As shown in Fig. 4K, L, the immune-relevant expression patterns clustered in C1 were associated with a worse patients’ outcome in the whole cohort encompassing all MPN subtypes, but not only in the PMF patients. Next, we aimed to link the allele frequencies of *JAK2* mutations in the BM (ranging from 11.1 up to 94.5%) with the expression of IFN-γ pathway components. However, only a trend of higher mutated allelic frequencies and increased expression of pSTAT1, but not of pJAK2, pSTAT3 or OAS1 was detected in the BMB (Fig. 4M–P).

**Fig. 5:**
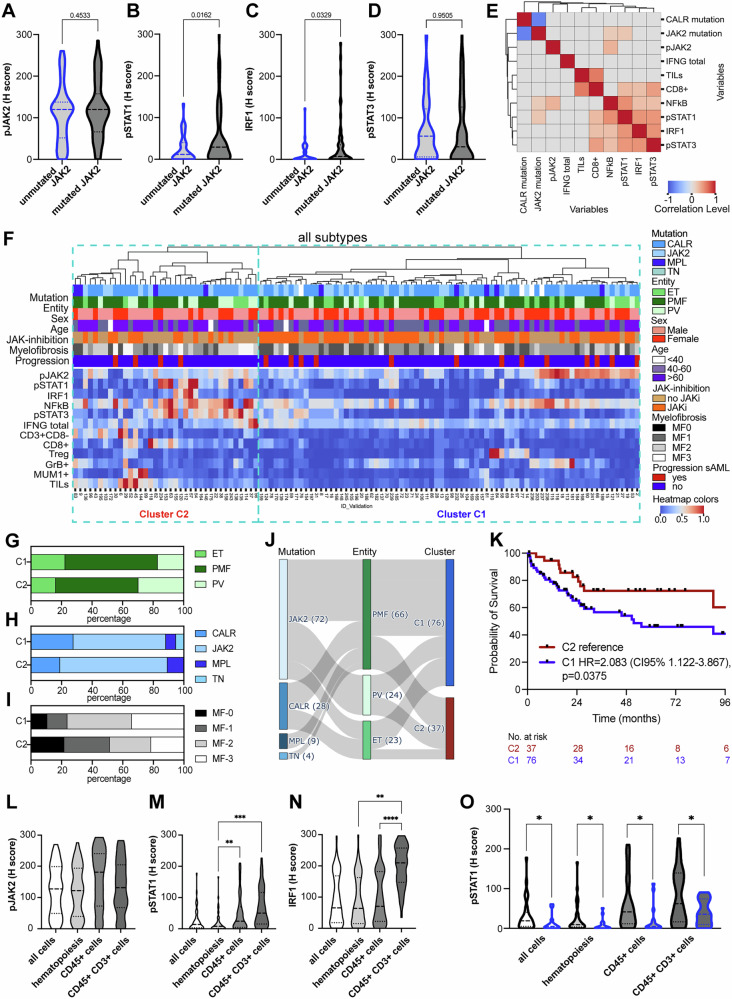
Analysis of the BM TME, MPN subtypes and survival in the validation cohort. **A**–**D** The expression of selected IFN-γ pathway components in MPN patients with *JAK2* versus *CALR* mutations. The p-values are given as numbers. **E** Cluster correlation map of the IFNG expression with JAK/STAT signaling pathway components and the immune cell composition in the BM. Pearson correlation coefficients are displayed by different colors defined in the scale bar, and only statistically significant correlations (*p* < 0.05) are depicted. **F** Unsupervised clustering based on the expression of components of JAK/STAT signaling, IFN-γ signaling, NF-kB and immune cell frequency is presented as a heatmap. Red tiles denote high expression, while blue tiles correspond to low expression (see color scheme heatmap at the right). The seven horizontal bars above the heatmap indicate the classification of samples according to driver mutation, MPN entity, sex, age, previous JAK inhibition prior to BMB, myelofibrosis and the progression to sAML in the follow up. The legend of the color labels is displayed on the right side of the heatmap. The clustering revealed two main clusters (C1-C2) based on the expression of the different factors. **G**–**I** Bar plots show the distribution of MPN entity, driver mutations, and degree of myelofibrosis. **J** Sankey plot shows the interrelationship of driver mutations, MPN entity, and immune clusters. **K** Kaplan-Meier estimators illustrate the prognostic significance of TME based clusters of MPN patients. **L**–**N** The expression of selected IFN-γ pathway components in MPN patients in all cells, hematopoietic cells, CD45^+^ cells and CD45^+^CD3^+^ cells, respectively. Significant differences are marked with an asterisk (**p < 0.001; ***p < 0.0001). **O** pSTAT1 expression in all cells, hematopoietic cells, CD45^+^ cells and CD45^+^CD3^+^ cells depending on the driver mutation (black border indicates a mutation in *JAK2* and blue borders indicate mutations in *CALR*, *MPL* or triple negative cases).

These findings were validated in another, independent MPN cohort encompassing 113 MPN BMB of patients with matched characteristics regarding age, sex and MPN entities, but enriched for MPN with mutations in *CALR* and *MPL*, demonstrating a higher expression of IFN-γ pathway components in *JAK2*-mutated MPN compared to MPN carrying *CALR* or *MPL* mutations as well as TN cases (Fig. 5A–D). In BMB of MPN patients with mutations in *JAK2*, a positive correlation with pSTAT1 and NF-kB expression was found. Higher pSTAT1 and IRF1 expression correlated with higher infiltration by CD8^+^ T cells (Fig. 5E). Unsupervised clustering revealed again two major immune clusters with similar characteristics as in the test cohort (Fig. 5F). The cluster C1 also demonstrated a lower expression of pSTAT1, pSTAT3, IRF1 and NF-kB as well as lower frequencies of different immune cell subsets, while the expression of pJAK2 was enriched in some patients of this cluster. Cluster C2 encompassed more frequently *JAK2*-mutated MPN, which was also associated with lower BM fibrosis and JAKi-naive cases (Fig. 5G–J). Analogous to the test cohort, patients in the cluster C2 had a significantly better survival (Fig. 5K). Furthermore, cell type specific expression analysis demonstrated the highest expression of pSTAT1 in CD45^+^ cells, including CD45^+^CD3^+^ T cells (Fig. 5L–N). Comparison of MPN samples with and without JAK2 mutation revealed a higher expression of pSTAT1 in JAK2 mutated cases within all cell subsets analyzed (Fig. 5O).

### Upregulation of ICP molecules and aberrant expression of classical and non-classical HLA-I molecules in MPN

**Fig. 6:**
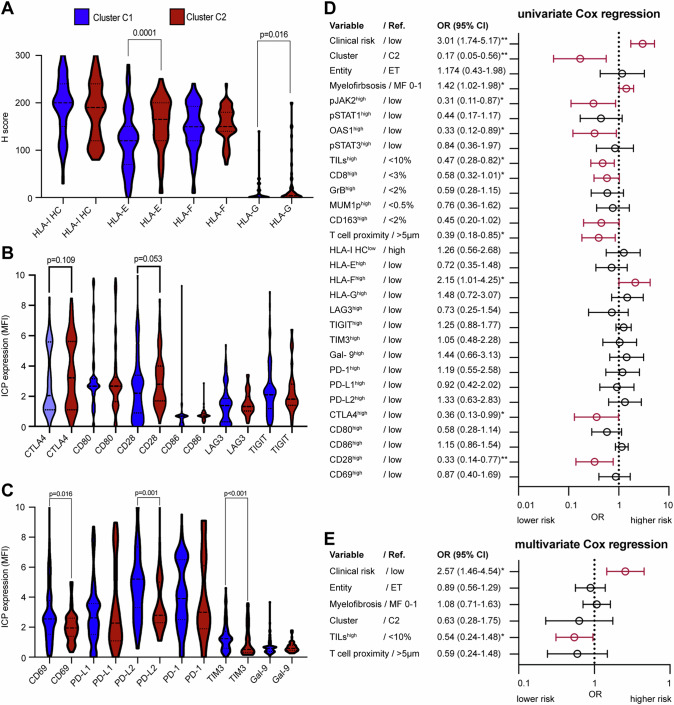
Altered immune cell infiltration, HLA-I, HLA-Ib and ICP expression in MPN and their prognostic significance. The surface expression of different HLA-I antigens and ICPs was determined by conventional IHC as well as by multispectral imaging. Differences in the expression in MPN diseases when compared to HC are depicted as box plots showing (**A**) HLA-I antigens and different ICP-molecules (**B**, **C**) in the immune clusters. The *p*-values are given as numbers. **D** Univariate cox regression analysis depicted as Forrest plots. The prognostic value of different immune response relevant factors was analyzed in 112 MPN patients of the test cohort. The hazard ratios and the 95% confidence interval are given. Significant factors are marked with an asterisk (*p* < 0.05). **E** Multivariate Cox regression analysis. Results are depicted in a Forrest plot. The hazard ratios and the 95%- confidence interval are given. Significant factors are marked with an asterisk (*p* < 0.05).

In order to investigate the expression of different immune modulatory molecules, like HLA-I antigens or ICPs, single and multiplex IHC staining were performed in the test cohort. A mean lower expression of HLA-I HC, HLA-E and HLA-F was detected in MPN subtypes compared to nnBM, whereas HLA-G was in average higher expressed in MPN samples (Supplementary Fig. S6A). Higher expression levels of the ICP molecules LAG3, PD-L1, TIM-3, Gal9, CTLA4, CD80, CD86 and CD28 as well as the T cell activation marker CD69, but not of PD-1 and PD-L2 were detected in MPN samples compared to nnBM (Supplementary Fig. S6B–D). The IFN-γ pathway component enriched cluster C2 showed a higher HLA-E and HLA-G, but not HLA-I HC or HLA-F expression. Furthermore, a trend of higher CTLA4 and CD28, but lower CD69, PD-L2 and TIM-3 expression was found in cluster C2 (Fig. 6A–C).

### Prognostic significance of the TME composition in combination with HLA-I and ICP expression

Alterations in the frequency and composition of immune cell infiltration and the expression of immune response-relevant molecules on tumor cells have clinical relevance in myeloid malignancies [35, 36]. Therefore, the prognostic value of the frequency and spatial distribution of TILs (T and B lymphocytes) as well as of HLA-I, HLA-Ib and ICP expression levels was analyzed in MPN and compared to the clinical risk scores (DIPPS score for PMF, European LeukemiaNet stratification for ET, and the IPSS for PV). Univariate cox regression analysis revealed that higher numbers of TILs, CD8^+^ T cells and GrB^+^ T cells, a close proximity of CD3^+^CD8^+^ and CD3^+^CD8^-^ T cell subsets and a higher expression of CTLA4, CD80 and CD28 was associated with a better patients’ survival. In contrast, HLA-I HC, HLA-E or HLA-G expression had no impact on the patients’ survival, while higher HLA-F expression levels were associated with an inferior outcome of MPN patients (Fig. 6D). However, the multivariate cox regression demonstrated that the number of TILs in the BMB is the only independent immune marker besides the clinical risk stratification associated with patients’ outcome (Fig. 6E) driving the prognostic value of the cluster C2.

## Discussion

MPN are characterized by a constitutive activation of JAK/STAT signaling pathway mediated by mutations in e.g., *JAK2* gene leading to the phosphorylation of STAT3 and activation of downstream effectors, which are crucial for disease initiation and progression [3, 6]. Activated *JAK2* is also linked to the consecutive activation of the IFN-γ signaling pathway accompanied by an increased expression of STAT1 that is essential for an adequate host immunity [7]. During the last two decades, IFN-γ signaling has been shown to play a central role not only in anti-tumor immune responses but also in immune evasion of malignant cells due to alterations of the surrounding TME [11]. There is growing evidence that chronic IFN-γ signaling inhibits T cell diversity to restrict anti-tumor immunity, while the inhibition of IFN-γ signaling leads to phenotypically less exhausted TILs, but impaired ICP inhibition [12]. Furthermore, the expression of classical HLA-I molecules, HLA-G and ICP molecules [14, 17], like PD-1 [16], PD-L1 [37], and LAG3 [38], is directly regulated by IFN-γ. Due to potential intrinsic activation of the IFN-γ signaling in MPN this study aimed to understand the interrelationship of this pathway with the local BM TME and its effects on the patients’ outcome.

This study demonstrated an activation of the IFN-γ signaling pathway in 265 MPN BMB in comparison to HCs (nnBM), which was confirmed by the analysis of several publicly available RNAseq data sets [39]. In addition, in vitro analysis of *JAK2* V6176F-mutated human cells revealed an intrinsic activation of the IFN-γ signaling pathway despite the lack of IFN-γ in the cell supernatants suggesting an influence of the genetic drivers. Bioinformatics analysis confirmed the activation of this central immunological pathway in MPN in comparison to HCs. Continuative comprehensive investigations of the expression pattern of IFN-γ signaling components in MPN BMB and publicly available MPN data sets as well as human *JAK2*- and *CALR*-mutated cell lines revealed a more pronounced activation of this immune pathway in *JAK2*-mutated than in *JAK2* wildtype MPN. Next, we tested whether IFN-γ signaling was also independent of the local expression of IFN-γ expression in vivo. However, *IFNG* mRNA was not only expressed by T cells, but also in hematopoietic cells as also reported by Steka and co-authors in AML cells before [39, 40]. BM mononuclear cells from AML patients with blast counts >80% demonstrated that IFN-γ was mainly secreted by more mature CD34^−^ CD33^+^ AML cells, but less commonly secreted by NK and T cells, which are generally known to express IFN-γ [40]. In depth analysis using MSI revealed a cell type specific expression in MPN BM cells with the highest expression of pSTAT1 and IRF1 in CD45^+^ cells, encompassing lymphocytes like B and T cells, but also in myeloid cells like monocytes, macrophages and granulocytes [41]. To validate these findings, we further analyzed a publicly available scRNAseq data set, which showed an upregulation of IFN-γ signaling mainly in myeloid cells, particularly in monocytes, but a downregulation of IFN pathways in erythropoietic cells. The significant different expression of this pathway in myeloid versus erythropoietic cells might be due to distinct regulatory mechanisms in these cells, such as e.g., a posttranscriptional control of IFN signaling components, but this hypothesis has still to be proven. However, it is noteworthy that in this data set, the number of erythropoietic cells is overrepresented encompassing more than 80% of all cells. Moreover, this upregulation of IFN-γ response in monocytes was independent from the local IFNG expression. The lack of IFN-γ expression and secretion in our in vitro cell models might be due to a context-specific expression of soluble factors. Such in vitro differences of the secretome of myeloid cells, e.g. macrophages, have been reported before depending on cultivation in neoplastic cell supernatants [42]. Moreover, phenotypically changes of neoplastic cells upon co-culture with immune subpopulations have also been shown [43]. Further, a lack of IFN-γ in our cell system could also be explained by the immaturity of the cultivated cells, while the MPN BM is usually dominated by more mature cells. In vivo, the increased expression of IFNG mRNA in the BMB correlated with an activated IFN-γ signaling in the hematopoiesis with an expression of IFN-γ signaling components in different hematopoietic cells, e.g., megakaryocytes and myelopoietic precursors. MPN arise from hematopoietic stem cells (HSC) that can differentiate into both myeloid and lymphoid hematopoietic lineages [44, 45]. *JAK2* V617F as the most common MPN driver mutation, has frequently been detected in myeloid-derived cells, like monocytes and macrophages, but rarely found in lymphocytes. This has been linked to an impaired lymphoid differentiation of *JAK2* V617F stem and progenitor cells [45]. Despite a cell subset-specific expression analysis of IFN-γ signaling components in mutated and non-mutated cells of the BM TME was not possible, we investigated whether the gene allele frequency of mutations in the *JAK2* gene affected the IFN-γ signaling activity within the BM in general and if so, whether this also influences the local BM TME composition. However, the mutated allele frequency of *JAK2* was not linked with a more pronounced activation of the IFN-γ signaling, since only a slightly higher expression of pSTAT1 was found in patients with higher levels of the mutated allele. Although no direct link between the allele frequencies of mutated JAK2 alleles in the BMB was found, differences in the composition of the BM TME between MPN samples with driver mutations in *JAK2* or *CALR/MPL* as well as TN cases were detected, which were associated with a lower expression of IFN- γ signaling components in *JAK2* wild type MPN. In general, the activation of the IFN-γ signaling pathway was positively associated with the density of the local immune cell infiltration and the expression of classical and non-classical HLA-I molecules as well as the ICPs CTLA4 and LAG-3, which has already been published [17], while an inverse correlation with PD-1/PD-L1 and PD-L2 was demonstrated. A downregulation of PD-1 by IFN-γ has been described before [16], while IFN-γ is known to upregulate the PD-L1 expression [37]. The slightly higher TIGIT expression in MPN with activated IFN-γ signaling contrasts with other reports linking that a higher expression levels to a decreased IFN-γ secretion in both healthy individuals and HIV infected patients [46, 47].

It is noteworthy that not only an intra-tumoral, but rather also an inter-tumoral heterogeneity in the expression of these immune relevant markers was found in the different MPN BMB. An unsupervised clustering model analyzing TILs and immune cell subsets as well as immune response-relevant molecules including IFN-γ signaling components revealed two distinct clusters, which were associated with MPN subtypes, BM fibrosis, JAKi and genetic drivers, but not with age or sex. A better survival outcome of MPN patients was observed in MPN patients in both the test and the validation cohort of cluster C2. Cluster C2 was characterized by a high expression of IFN-γ signaling components and NF-kB as well as a higher immune cell infiltration, which is in line with the described survival benefits of patients with myeloid malignancies exhibiting an activated immune TME [35, 36, 48]. In general, a favorable prognostic value for different immune response-relevant variables was found, including higher TIL frequency, CD8^+^ T cell infiltration, increased spatial proximity of T cell subpopulations as well as the expression of the ICP axis CTLA4/CD80/CD86/CD28. In contrast, patients with a high HLA-F expression in the BM showed a shorter PFS. However, multivariate Cox regression analysis including the immune marker expression-based clusters revealed the TIL frequency as the strongest predictive marker, which might also explain the better survival of MPN patients in the cluster C2. In contrast, the IFN-γ signaling itself had no significant independent prognostic value.

In conclusion, an activation of the IFN-γ signaling pathway was predominantly detected in *JAK2*-mutated MPN, which was associated with BM fibrosis and alterations in the local immune cell infiltration of the BM TME as well as the expression of some immune relevant molecules including NF-kB, HLA-Ib and ICPs. An enriched IFN-γ signaling was found throughout different disease stages, with the highest expression in white blood cells, particularly T cells and monocytes, which was associated with TIL infiltration. Survival analysis revealed that the frequency of TILs in the BM was the strongest predictive immune marker in MPN, independent of the clinical risk and the degree of BM fibrosis. These data suggest a complex interaction of the neoplastic hematopoiesis with the surrounding TME in MPN that drives disease progression and BM fibrosis. A limitation of this study is the lack of knowledge about the cell subtype-specific presence and spatial distribution of the molecular drivers in neoplastic hematopoiesis, including the local immune cell infiltrate, which could provide deeper insights into the immunological regulation in MPN. Further studies are urgently needed to understand the complex underlying mechanisms of the interaction of neoplastic and non-neoplastic cells in MPN and its dependence on driver mutations. A better understanding of these interactions and the role of the IFN-γ signaling in MPN will pave the rational for the development of novel treatment options for this disease.

## Supplementary information

Supplementary Table S1

Supplementary Table S2

Supplementary Figure Legends

Supplementary Figure S1

Supplementary Figure S2

Supplementary Figure S3

Supplementary Figure S4

Supplementary Figure S5

Supplementary Figure S6

## Data Availability

The data generated in this study are available upon request from the corresponding author.
