# Supplementary material for: Association of the composition of the bone marrow tumor microenvironment in BCR::ABL1-negative myeloproliferative neoplasms with IFN-γ signaling and driver mutations
Source: Leukemia. 2025 Aug 5;39(10):2391–405. doi: 10.1038/s41375-025-02706-3 (PMC12463677; doi:10.1038/s41375-025-02706-3)
Supplement: Supplementary file 1 — Supplementary Table S1 [file 41375_2025_2706_MOESM1_ESM.docx]

**Supplementary Table S1: Overview of the antibodies applied for IHC and MSI.**

| **Antibody** | **Company** | **Clone** |
| --- | --- | --- |
| CD3 | Labvision, Germany | SP7 |
| CD8 | Abcam, UK | SP16 |
| CD28 | Abcam, UK | ab 243228 |
| CD33 | Leica Biosystems, Germany | PWS44 |
| CD34 | Thermo Fisher Scientific | QBend |
| CD45 | CD45 | 2B11 + PD7/26 |
| CD68 | Dako, USA | PG-M1 |
| CD69 | Abcam, UK | ab233396 |
| CD71 | Cell Marque, USA | MRQ-48 |
| CD80 | Abcam, UK | ERP1157(2) |
| CD86 | Cell signaling, UK | E2G8P |
| CD117 | Dako, USA | CD117 |
| CD163 | Cell marque, USA | MRQ-25 |
| CTLA-4 | Medac, Germany | Tinto CD152 |
| FOXP3 | Abcam, UK | 236A/E7 |
| Gal-9 | Cell signaling, UK | D9RA4 |
| GAPDH | Cell signaling, UK | 14C10 |
| GrB | Cell marque, USA | 262A-14 |
| HC-10 | Bioss, USA | HSP09-35 |
| HLA-E | Biozol, Germany | LS‑B2857 |
| HLA-F | Thermo Fisher Scientific | JE54-04 |
| HLA-G | Abcam, UK | ab52455 |
| IRF1 | Cell signaling, UK | D5E4 |
| JAK1 | Cell signaling, UK | 6G4 |
| JAK2 | Cell signaling, UK | D2E12 |
| LAG3 | Leica Biosystems, Germany | 12H6 |
| Lysozyme | Epitomics, USA | EP134 |
| MPO | Dako, USA | A0398 |
| MUM1p | Dako, USA | MUM1p |
| OAS1 | Abcam, UK | ab232862 |
| PD-L1 | Zytomed, Germany | CAL10 |
| PD-L2 | Cell signaling, UK | D7U8C |
| PD-1 | Zytomed, Germany | NAT105 |
| pJAK1 | Cell signaling, UK | Y1034/1035 |
| pJAK2 | Abcam, UK | phospho JAK2 Y1007 + Y1008 |
| pSTAT1 | Cell signaling, UK | Tyr701 |
| pSTAT3 | Cell signaling, UK | tyr705 |
| STAT1 | Cell signaling, UK | 42H3 |
| STAT3 | Cell signaling, UK | 124H6 |
| TIGIT | Biozol, Germany | USC-PAN056 HU01-10 |
| TIM-3 | Abcam, UK | Ab241332 |
