# Supplementary material for: Association of the composition of the bone marrow tumor microenvironment in BCR::ABL1-negative myeloproliferative neoplasms with IFN-γ signaling and driver mutations
Source: Leukemia. 2025 Aug 5;39(10):2391–405. doi: 10.1038/s41375-025-02706-3 (PMC12463677; doi:10.1038/s41375-025-02706-3)
Supplement: Supplementary file 2 — Supplementary Table S2 [file 41375_2025_2706_MOESM2_ESM.docx]

**Supplementary Table 2: Overview of the multispectral imaging (MSI) panels.**

| **Panel number** | **Cohort analyzed** | **Antibodies** |
| --- | --- | --- |
| Panel-1 | Test and validation cohort | CD3, CD8, FOXP3, MUM1p, CD34 and granzyme B (GrB) |
| Panel-2 | Test cohort | CD3, CD34, PD-1, PD-L1 and PD-L2 |
| Panel-3 | Test cohort | CD3, CD8, CD11c, CTLA-4, CD80 and CD86 |
| Panel-4 | Test cohort | CD68, CD163, CD16, CD56, TIM-3 and Gal-9 |
| Panel-5 | Test cohort | CD3, LAG-3, TIGIT, CD28, CD69 and CD33 |
| Panel-6 | Test and validation cohort | pJAK2, CD3, CD45, pSTAT1, IRF1 and OAS1 |
