# Supplementary material for: Association of the composition of the bone marrow tumor microenvironment in BCR::ABL1-negative myeloproliferative neoplasms with IFN-γ signaling and driver mutations
Source: Leukemia. 2025 Aug 5;39(10):2391–405. doi: 10.1038/s41375-025-02706-3 (PMC12463677; doi:10.1038/s41375-025-02706-3)
Supplement: Supplementary file 3 — Supplementary Figure Legends [file 41375_2025_2706_MOESM3_ESM.docx]

**Supplementary Figure S1:** (A) IFN-γ-regulated genes in healthy BM and lymphoid tissues and MPN. The 91 genes were selected based on the open-source, peer-reviewed pathway database reactome (<https://reactome.org>, ID: R-HSA-877300.6). Their expression was visualized with a heatmap using the publicly available expression data of different tissues (bone marrow, tonsil, lymph node, and spleen) of reactome database. (B) ssGSEA for IFN-γ response genes in different human hematopoietic cell lines. The NES is separately shown for each cell line and the available genes of the IFN-γ response gene set (MSigDB_Hallmark_2020) are given in the bar plot. The colors of the bars show the presence or absence of genetic aberrations of the *JAK2* gene. (C) GSEA of the *JAK2*-mutated HEL und SET-2 cells versus *CALR*-mutated MARIMO cells using the MSigDB_Hallmark_2020 gene set. The NES is shown with bars and the colors of the bars represent the FDR as shown in the legend. (D-G) Immunohistochemical expression of IFN-γ signaling components are shown in patients with low (MF-0-1) and overt (MF-2-3) BM fibrosis. (H-I) Box plots showing the expression of OAS1 and IRF1 in *JAK2*-mutated MPN versus wild type JAK2 MPN. (J) Box plot demonstrating the allele frequencies of mutated *JAK2* and *CALR* genes in the BM. (K) GSEA of the GSE174060 data set of IFN-γ response genes (MSigDB_Hallmark_2020) comparing the transcriptome of CD34^+^ cells from MPN versus HC.

**Supplementary Figure S2:** (A) Comparison of the expression (log2FC) of different immune genes in the GSE206768 data set of PMF cases with MF-3 versus MF-0. Significantly upregulated genes (p<0.05) are depicted in a volcano plot and highlighted in red including IL-8 and TGF-ß. (B) GSEA (MSigDB_Hallmark_2020) of MPN samples with MF-3 versus MF-0 shown as a bar graph. The NES is given as the value of the bars and the FDR is depicted in different colors and gene sets with a p-value <0.05 are written with bold letters. (C) GSEA of the IFN-γ response (MSigDB_Hallmark_2020) comparing CD34+ cells of MPN with overt BM fibrosis (MF-3) versus low fibrosis (MF-0). (D-E) Violin plots show the expression of IFNG and CD4 mRNA in samples with low (MF 0) and high (MF 3) myelofibrosis. Significant difference is marked with asterisks (** p<0.001). (F-H) Scatter plots show an association of IFNG mRNA expression with STAT1, STAT3 and CD4 mRNA expression, respectively.

**Supplementary Figure S3:** Analysis of the IFN-γ response genes in public data sets GSE103176 (left) and GSE174060 (right). (A-B) PCA analysis with *JAK2*-mutated cases depicted in red and *CALR*- or *MPL*-mutated cases in blue. (C-D) GSEA using the MSigDB_Hallmark_2020 gene set. Significantly up-/downregulated gene sets are highlighted with bold letters. (E-F) Heatmaps of the IFN-γ response gene set according to the MSigDB_Hallmark_2020 gene set. (G-H) GSEA depiction of the IFN-γ response genes.

**Supplementary Figure S4:** Correlation map of different immune-response relevant markers and components of the JAK/STAT signaling pathway. The correlation coefficients are depicted in different colors and the Pearson correlation coefficient is given. Red tiles denote a positive correlation, while blue tiles correspond to an inverse correlation. Significant associations (p<0.05) are given as bolt numbers of the correlation coefficient.

**Supplementary Figure S5:** Analysis of IFN-γ response genes in a publicly available scRNAseq data set (29). Violin plots show the expression of STAT1, IRF1 and IFNG in distinct cell types in HC and MPN. Significant differences between HC and MPN are labeled with p-values.

**Supplementary Figure S6:** Box plot depiction of the HLA-I antigens and ICP expression in ET, PMF and PV versus HC. The p-values are given as asterisk (*<0.05, **<0.001, ***<0.0001).
