# Supplementary figures and images for: Association of the composition of the bone marrow tumor microenvironment in BCR::ABL1-negative myeloproliferative neoplasms with IFN-γ signaling and driver mutations
Source: Leukemia. 2025 Aug 5;39(10):2391–405. doi: 10.1038/s41375-025-02706-3 (PMC12463677; doi:10.1038/s41375-025-02706-3)

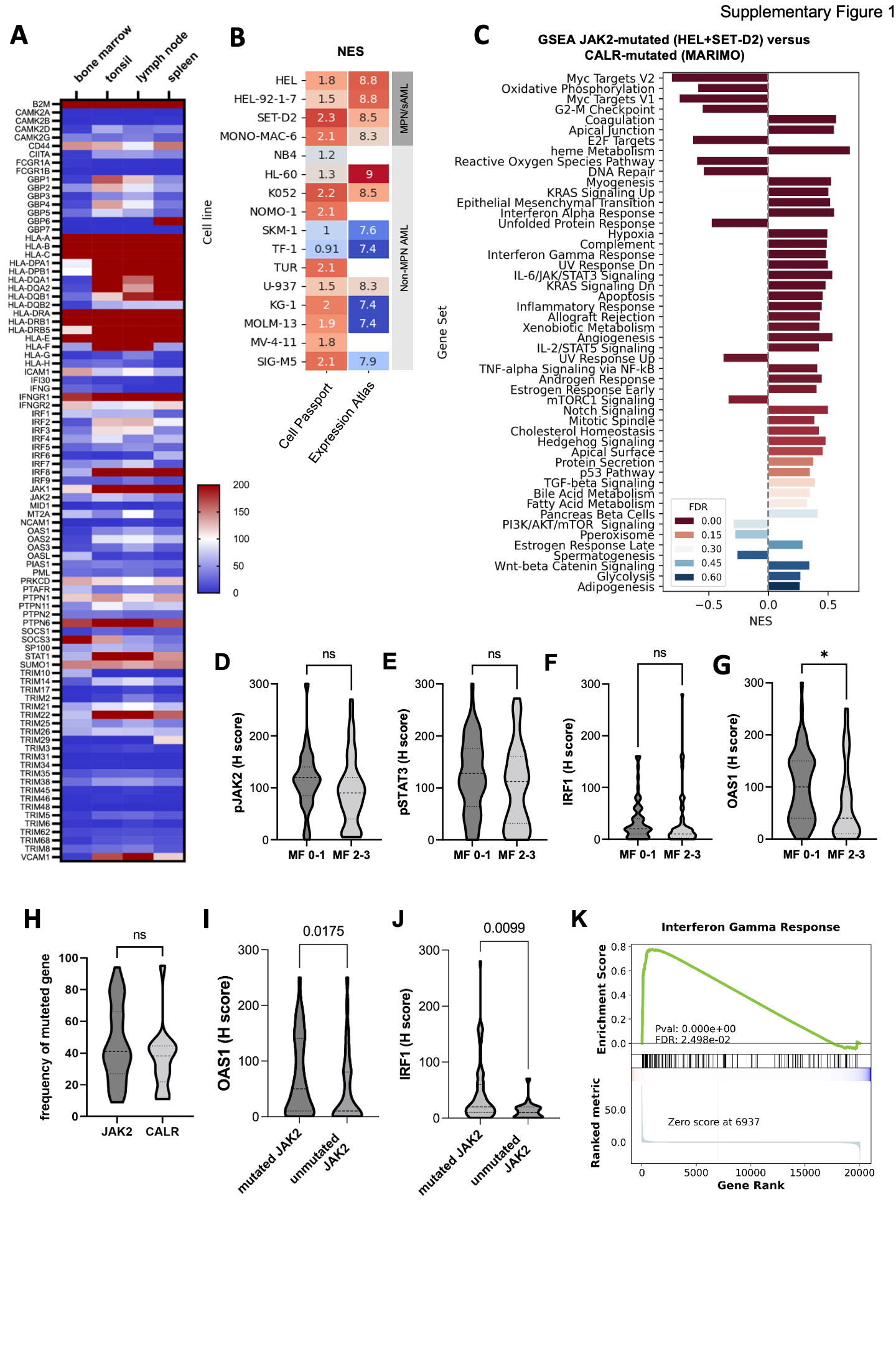

Supplement: Supplementary file 4 — Supplementary Figure S1 [file 41375_2025_2706_MOESM4_ESM.png]

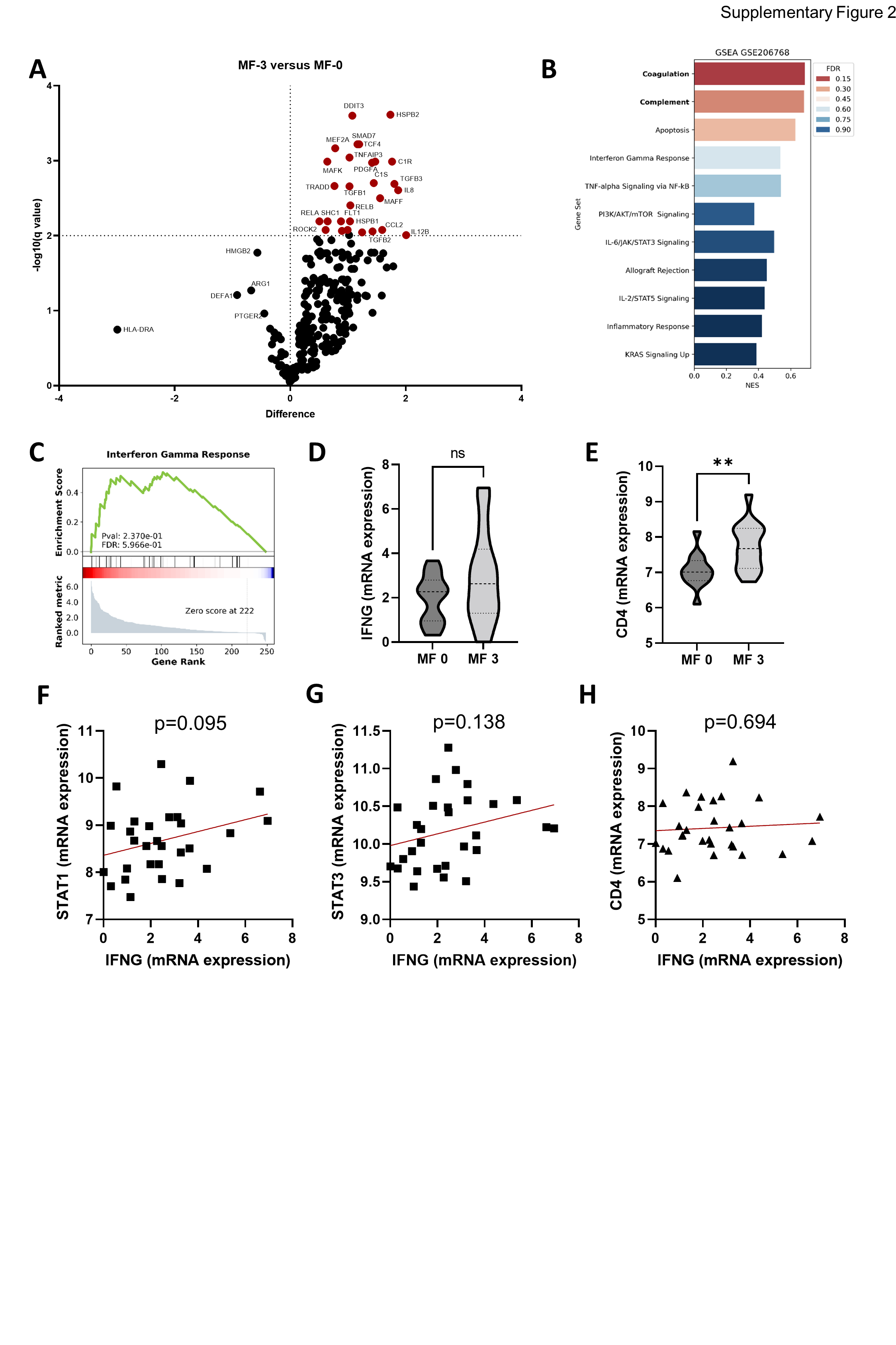

Supplement: Supplementary file 5 — Supplementary Figure S2 [file 41375_2025_2706_MOESM5_ESM.png]

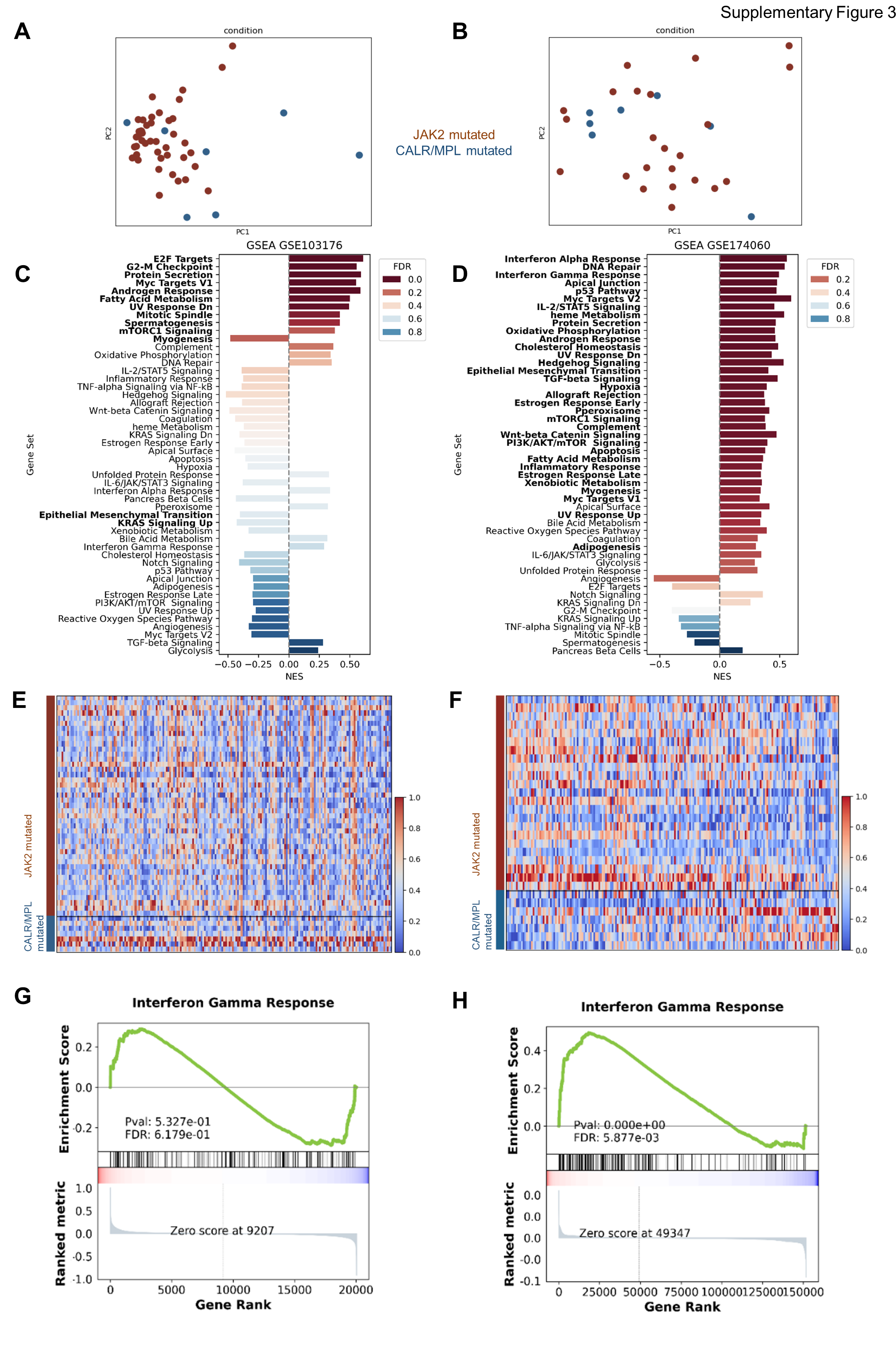

Supplement: Supplementary file 6 — Supplementary Figure S3 [file 41375_2025_2706_MOESM6_ESM.png]

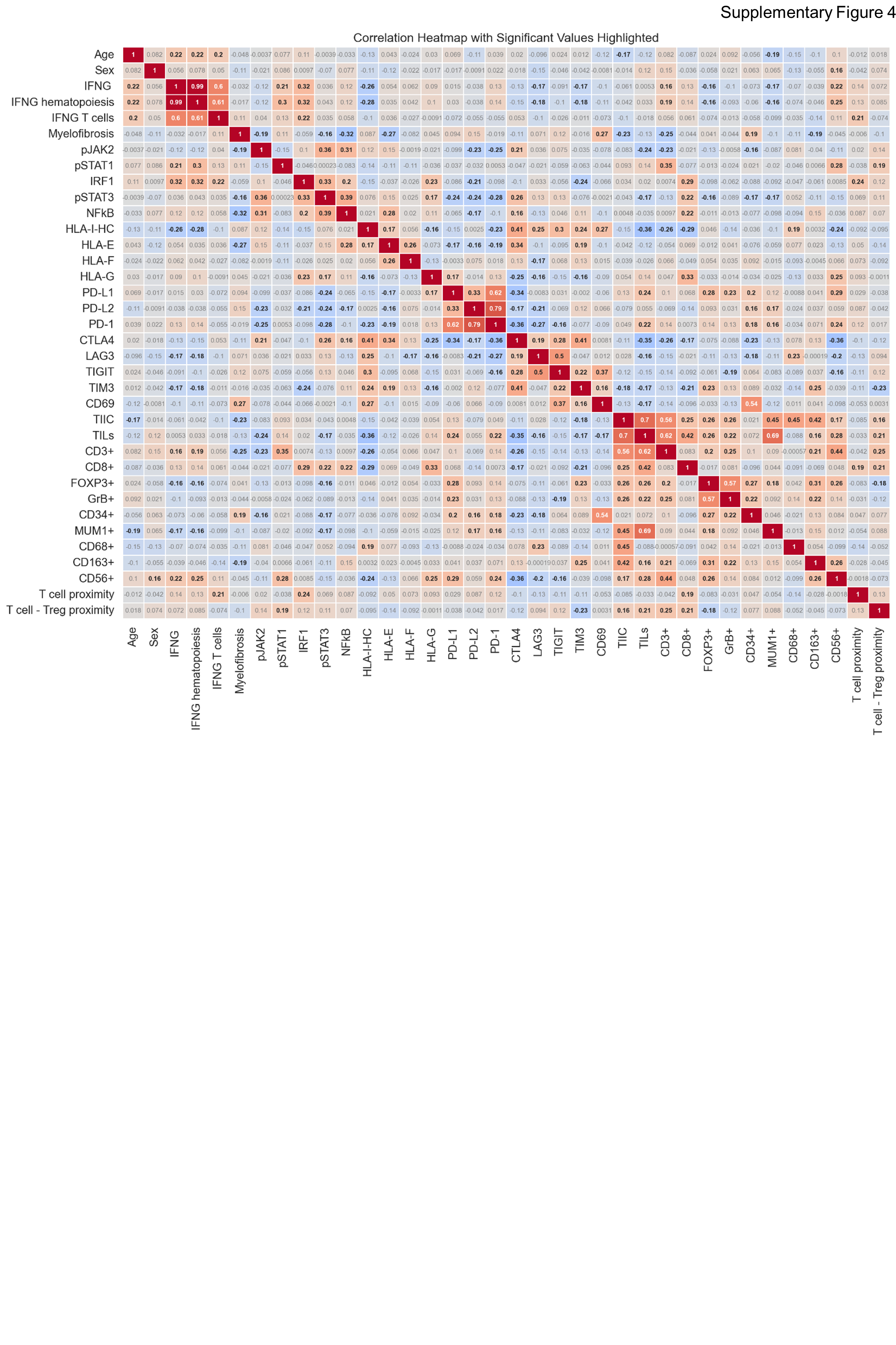

Supplement: Supplementary file 7 — Supplementary Figure S4 [file 41375_2025_2706_MOESM7_ESM.png]

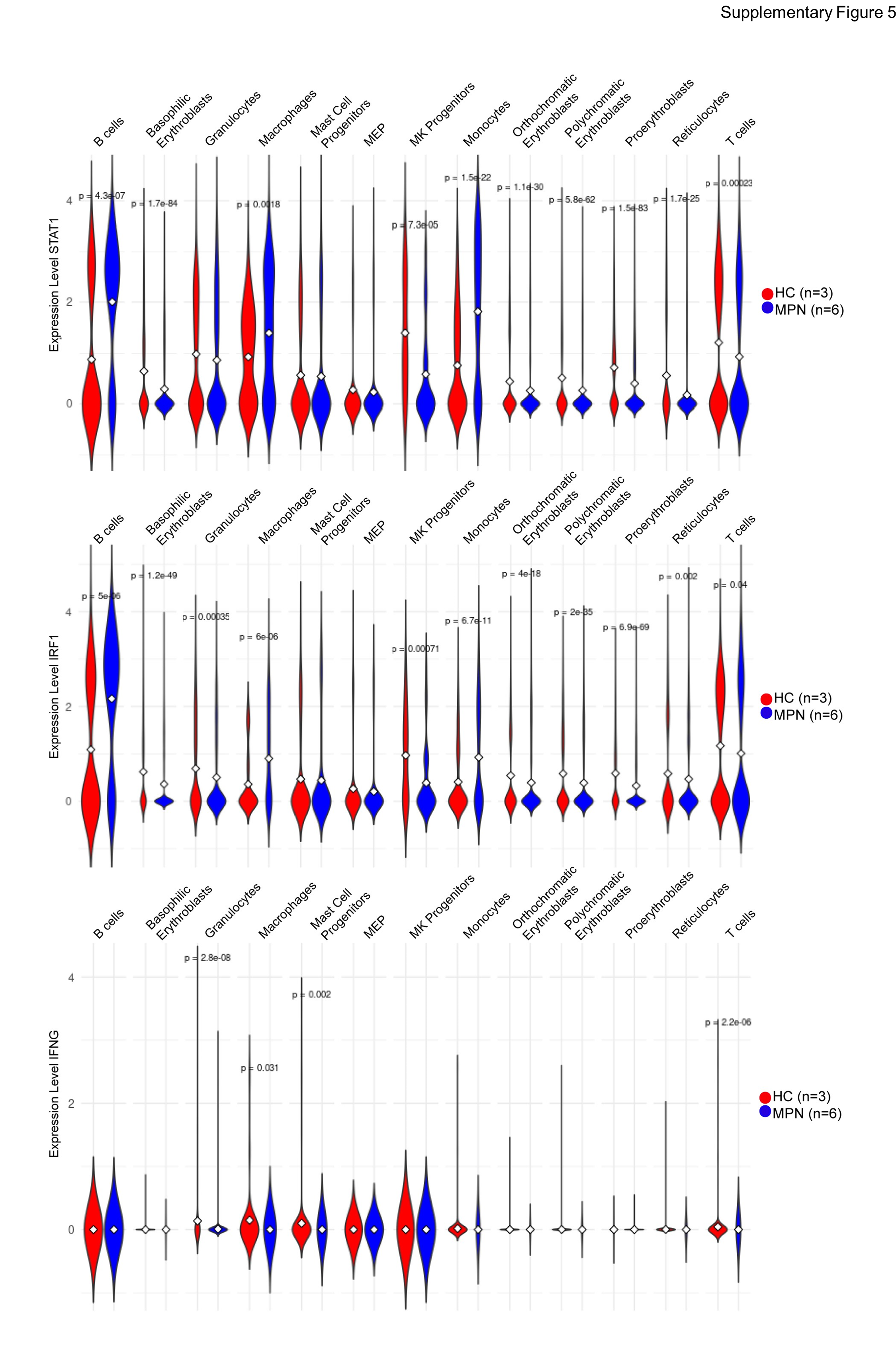

Supplement: Supplementary file 8 — Supplementary Figure S5 [file 41375_2025_2706_MOESM8_ESM.png]

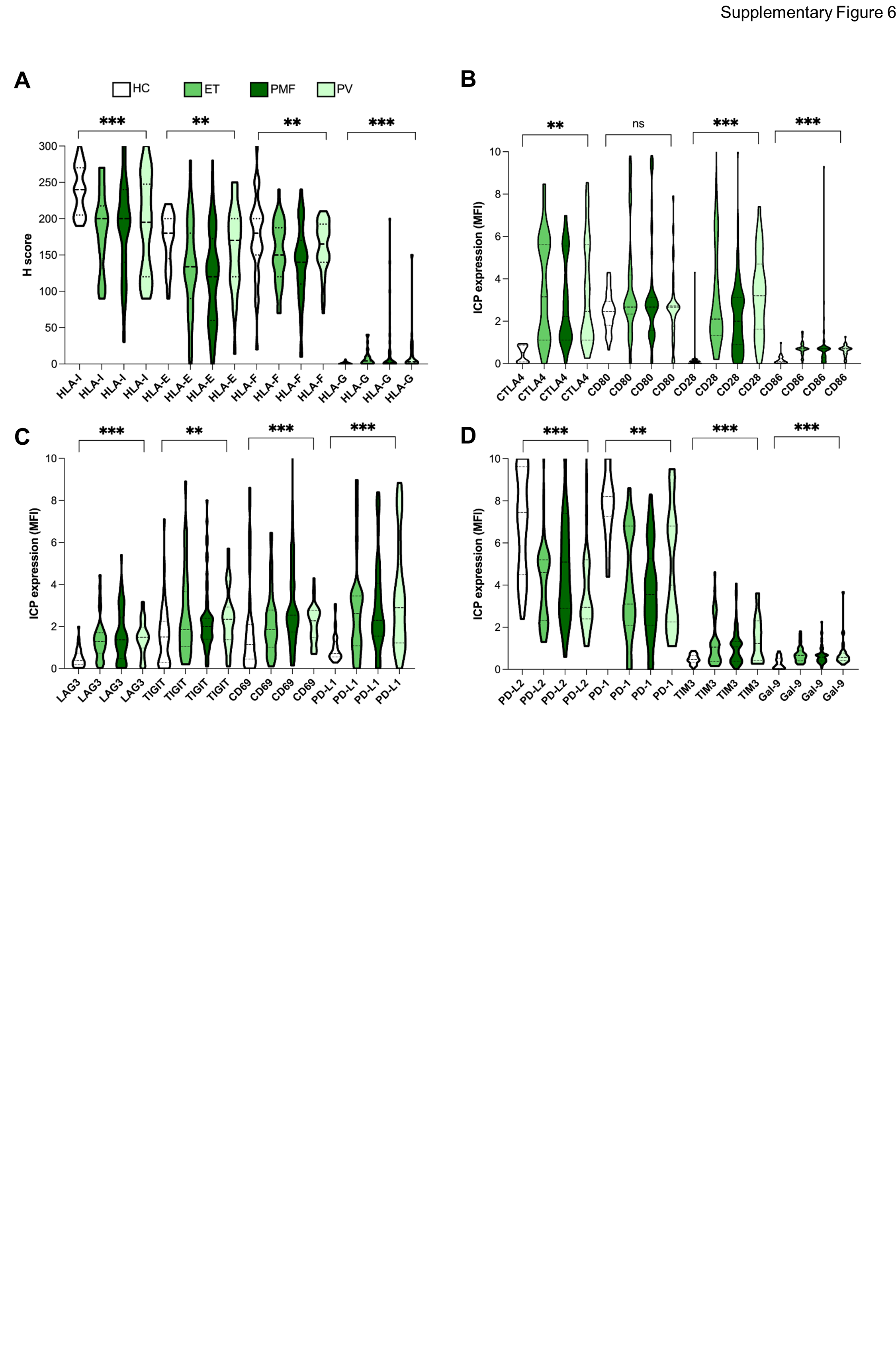

Supplement: Supplementary file 9 — Supplementary Figure S6 [file 41375_2025_2706_MOESM9_ESM.png]
